# Passive Surveillance of *Ixodes scapularis* (Say), Their Biting Activity, and Associated Pathogens in Massachusetts

**DOI:** 10.1089/vbz.2015.1912

**Published:** 2016-08-01

**Authors:** Guang Xu, Thomas N. Mather, Craig S. Hollingsworth, Stephen M. Rich

**Affiliations:** ^1^ Laboratory of Medical Zoology, Department of Microbiology, University of Massachusetts, Amherst, Massachusetts.; ^2^ RI Center for Vector-Borne Disease, University of Rhode Island, Kingston, Rhode Island.

**Keywords:** *Anaplasma phagocytophilum*, *Babesia microti*, *Borrelia burgdorferi*, *Ixodes scapularis*, Lyme disease, Massachusetts, Surveillance, Ticks, Tick-bite

## Abstract

A passive surveillance of tick-borne pathogens was conducted over a 7-year period (2006–2012), in which a total of 3551 ticks were submitted to the University of Massachusetts for PCR testing. The vast majority of these ticks were *Ixodes scapularis* from Massachusetts (*N* = 2088) and hence were the focus of further analysis. Two TaqMan duplex qPCR assays were developed to test *I. scapularis* ticks for the presence of three human pathogens: *Borrelia burgdorferi*, *Anaplasma phagocytophilum*, and *Babesia microti*. *I. scapularis* submissions were concentrated from Cape Cod, the eastern half of the state outside of the Boston metropolitan area, parts of Franklin and Hampshire counties along the Quabbin Reservoir watershed, and southwestern Berkshire county. Differences in seasonal activity pattern were observed for different developmental stages of *I. scapularis*. The largest proportion of tick bite victims were age 9 years and under. Nymphal ticks were found more often on lower extremities of their hosts, while more adult ticks were found on the head. Overall infection rate of *B. burgdorferi*, *A. phagocytophilum*, and *B. microti* in human-biting ticks was 29.6%, 4.6%, and 1.8%, respectively. *B. burgdorferi*-infected ticks were widely distributed, but *A. phagocytophilum*- and *B. microti*-infected *I. scapularis* were found mainly in the eastern half of the state. We found that 1.8%, 1.0%, and 0.4% of ticks were coinfected by *B. burgdorferi* and *A. phagocytophilum*, *B. burgdorferi* and *B. microti*, and *A. phagocytophilum* and *B. microti*, respectively, and 0.3% of ticks had triple coinfection.

## Introduction

The black-legged tick, *Ixodes scapularis*, transmits the bacterium *Borrelia burgdorferi*, which causes Lyme borreliosis, the most commonly reported arthropod-borne illness in the United States (Centers for Disease Control and Prevention 2015). These same ticks are also important vectors for other human diseases, including anaplasmosis and babesiosis. *I. scapularis* is well established throughout New England (Walk et al. 2009) and is generally not locally limited by host abundance (Guerra et al. 2002). The presence and abundance of *I. scapularis* are associated with soils, vegetation, and other environmental factors (Nicholson and Mather 1996, Guerra et al. 2002). As a result, the geographic distribution of these ticks and the pathogens they carry are not uniform, but patchy and discontinuous. Geographic information system (GIS) maps of tick and tick-borne pathogen distributions provide crucial information for awareness, prevention, and prediction of tick-borne diseases (Daniel et al. 2004).

Risk of tick-borne disease is not only associated with tick populations but also with preventative human behaviors. Early tick detection and removal dramatically decrease the chance of contracting a tick-borne disease when a tick bite occurs. However, approximately 70% of people who contract Lyme borreliosis do not recall being bitten (Poland 2001). Knowledge of tick attachment preferences (Felz and Durden 1999), duration of attachment (Piesman et al. 1987), and age-specific prevalence of transmission events (Bacon et al. 2008) is critical for increasing personal precautions and public health awareness.

Labor-intensive multisite tick flagging (Mather et al. 1996, Bunnell et al. 2003, Walk et al. 2009) and host trapping (Rand et al. 2003), the traditional methods of tick surveillance, are frequently performed only in areas of high tick density. Canine serosurveys are another method of active Lyme borreliosis surveillance to help predict human disease risk (Hinrichsen et al. 2001, Stone et al. 2005). Active surveillance methods generally do not link information about ticks and tick-borne diseases directly to human–tick encounters and often provide information on a limited geographic scale.

Passive tick surveillance can provide specific information about human–tick encounters and the incidence of tick-borne diseases on a large geographic scale. Although passive tick surveillance has been conducted in New York (Falco and Fish 1988), Maine (Rand et al. 2007), Canada (Ogden et al. 2006, Nelder et al. 2014), and by the U.S. military (Stromdahl et al. 2001), none has been performed in Massachusetts, a high-risk area for Lyme borreliosis. Additionally, limited pathogen infection data are available specifically for ticks parasitizing humans.

We examined the distribution of ticks and tick-borne pathogens, *B. burgdorferi*, *Anaplasma phagocytophilum*, and *Babesia microti*, in Massachusetts using GIS maps and PCR-based screening of total genomic DNA isolated from *I. scapularis*. We analyzed seasonal trends in the incidence of attacks by three different tick developmental stages. We also examined attachment sites of different stages of *I. scapularis* on human hosts as well as sex- and age-specific prevalence of tick bites at different attachment sites on human hosts. Results of our 7-year study demonstrate the value of passive tick surveillance in helping to understand the epidemiology of tick-borne diseases and provide valuable data for assessing the risk of Lyme borreliosis in Massachusetts.

## Materials and Methods

### Collection of ticks

Ticks were collected from July 2006 through December 2012 by offering a tick identification and *B. burgdorferi*, *A. phagocytophilum*, and *B. microti* detection service to the public through the University of Massachusetts Extension website (www.umass.edu/tick). We received tick specimens through postal mail at the University of Massachusetts Amherst enclosed in small plastic vials or zipper-locking bags. All persons submitting ticks were asked to complete a form indicating the location and date of tick collection; age, gender, and species of the host; and attachment site of the tick on the host's body.

### Tick identification

Preliminary species-level identification of each tick was based on published identification keys (Keirans and Clifford 1978, Keirans and Litwak 1989, Keirans et al. 1996). Ticks were categorized by developmental stage (larva, nymph, or adult) and engorgement levels (unengorged and engorged). Total DNA was extracted from each tick using Epicenter Master Complete DNA and RNA Purification Kits (Epicenter Technologies, Madison, WI) following the manufacturer's protocols and dissolved in 30 μL H_2_O. To determine the quality of DNA extraction and to verify the tick species, we amplified a fragment of the tick mitochondrial 16S rRNA gene using the primers shown in Table 1. Amplification reactions were performed in 25 μL volumes containing 1 μL DNA, 5 μL 5× buffer, 4 μL 25 μM MgCl_2_, 1 μL 10 mM dNTPs, 1 μL 10 μM each primer, and 0.2 μL 5 U/μL Taq polymerase (Promega, Madison, WI) using the Eppendorf ep mastercycler (Eppendorf, Westbury, NY) with the following program: 94°C for 1 min, and 40 cycles at 94°C for 15 s, 50°C for 15 s, and 72°C for 40 s. Amplified products were cleaned with the ExoSAP-IT kit (USB, Cleveland, Ohio) and then sequenced bidirectionally on an ABI 3130XL Genetic Analyzer (Applied Biosystems, Foster City, CA). Sequences were aligned and compared with tick 16S RNA reference sequences to verify the species of each sample (*Amblyomma americanum*; and L34296; *Dermacentor variabilis*; L34313; GenBank Acc. No. L43877; *I pacificus*; *I. scapularis* strain Florida; L43857; *I. scapularis* strain Massachusetts; L34300).

**Table 1.** TaqMan Duplex Assays to Detect Tick DNA, *Borrelia burgdorferi*, *Anaplasma phagocytophilum*, and *Babesia microti*

*Duplex*	*Target*	*Gene*	*Type*	*Sequences (5′-3′)*	*Con. (nM)*
1	Tick	16S	Forward	AATACTCTAGGGATAACAGCGTAATAATTTT	300
			Reverse	CGGTCTGAACTCAGATCAAGTAGGA	300
			Probe	FAM - AAATAGTTTGCGACCTCGATGTTGGATTAGGAT - BHQ1	125
			Standard Curve	Y = −3.579^*^LOG(X) +49.68, Eff. = 90.3%, RSq = 99.4%	
	Borrelia	23S	Forward	CGAGTCTTAAAAGGGCGATTTAGT	700
			Reverse	GCTTCAGCCTGGCCATAAATAG	700
			Probe	HEX-AGATGTGGTAGACCCGAAGCCGAGTG - BHQ1	300
			Standard Curve	Y = −3.305^*^LOG(X) +32.55, Eff. = 100.7%, RSq = 99.3%	
2	Babesia	Tubulin	Forward	GATTTGGAACCTGGCACCATG	700
			Reverse	AATGACCCTTAGCCCAATTATTTCC	700
			Probe	FAM - ATCTGGCCCATACGGTGAATTGTTTCGC- BHQ1	250
			Standard Curve	Y = −3.677^*^LOG(X) +20.71, Eff. = 87.1%, RSq = 99.8%	
	Anaplasma	MSP2	Forward	ATGGAAGGTAGTGTTGGTTATGGTATT	700
			Reverse	TTGGTCTTGAAGCGCTCGTA	700
			Probe	HEX- TGGTGCCAGGGTTGAGCTTGAGATTG- BHQ1	250
			Standard curve	Y = −3.628^*^LOG(X) +19.66, Eff. = 88.6%, RSq = 99.7%	

We performed TaqMan real-time PCR assays in two duplex formats with 20 μL reaction volumes using the Brilliant II QPCR Master Mix in a Stratagene MX3000P QPCR System. The cycling conditions included an initial activation of the Taq DNA polymerase at 95°C for 10 min, followed by 40 cycles of 15-s denaturation at 95°C, and 1-min annealing extension at 60°C.

### Detection of pathogen DNA in I. scapularis by TaqMan real-time PCR

Table 1 shows the probes and primers used in our real-time PCR assays. We used previously described primers and probes to detect *B. burgdorferi* and *A. phagocytophilum* (Courtney et al. 2004) and a new assay for *B. microti* with tick DNA detection as an internal control. The previously described assay was specific for *Borrelia burgdorferi* sensu lato and the analytical sensitivity was 50 borrelia spirochetes. The *Babesia* assay was specific for *B. microti* and the assay sensitivity was 40 copies of *B. microti* tubulin gene. We performed TaqMan real-time PCR assays in two duplex formats with 20 μL reaction volumes using the Brilliant II qPCR Master Mix (Agilent, La Jolla, CA) in a Stratagene MX3000P qPCR System. The first duplex detected tick DNA and *B. burgdorferi*, and the second duplex detected *A. phagocytophilum* and *B. microti*. In the first duplex, a probe that hybridizes to the 16S mtDNA gene in all hard tick species was used as an internal control. Cycling conditions included an initial activation of the Taq DNA polymerase at 95°C for 10 min, followed by 40 cycles of 15-s denaturation at 95°C, and 1-min annealing extension at 60°C.

## Results

### Geographical and seasonal distributions of tick species

We received a total of 3551 ticks representing seven species from July 2006 through December 2012. Among them, we identified 3127 *I. scapularis*, 231 *D. variabilis*, 159 *A. americanum*, 26 *I. pacificus*, 5 *Rhipicephalus sanguineus*, 1 *D. occidentalis*, and 1 *Haemaphysalis leporispalustris*. The canonical host of all three pathogens screened in our study is *I. scapularis*.

**FIG. 1.**
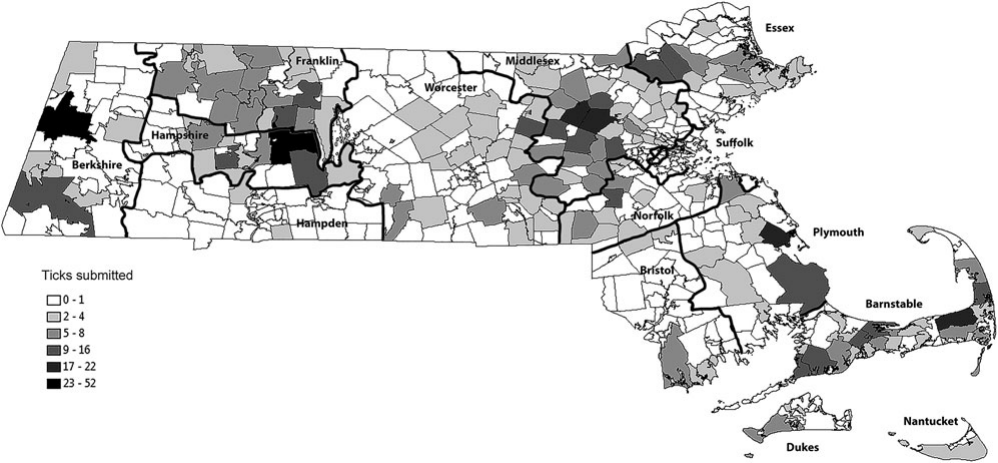
Distribution of 2088 *Ixodes scapularis* submissions in Massachusetts (2006–2012). The broadly defined high tick encounter areas are Cape Cod, the eastern half of the state outside of Suffolk county, parts of Franklin and Hampshire counties along the Quabbin Reservoir watershed, and in southwestern Berkshire county.

Of 3551 submitted ticks, 2203 (62.1%) originated from Massachusetts, comprising three species: 2088 *I. scapularis*, 108 *D. variabilis*, and 7 *A. americanum*. We received at least one tick from 292 of 359 incorporated towns (81%) in the state. Only 8 ticks were received from Martha's Vineyard and Nantucket island. Analyses of the geographic and seasonal distribution of ticks and three associated pathogens was restricted to *I. scapularis* found on humans in Massachusetts where we had tractable sample sizes. The number of *I. scapularis* submissions from Massachusetts increased annually: from 90 in 2006 to 570 in 2012. The numbers of submitted ticks correlated significantly with the cumulative Lyme borreliosis cases (Massachusetts Department of Public Health surveillance data) reported at the county level (*n* = 14 counties, *r*^2^ = 0.33, *p* < 0.05). Figure 1 shows the geographic distribution of the total *I. scapularis* ticks submitted from within Massachusetts over the period 2006–2012.

**FIG. 2.**
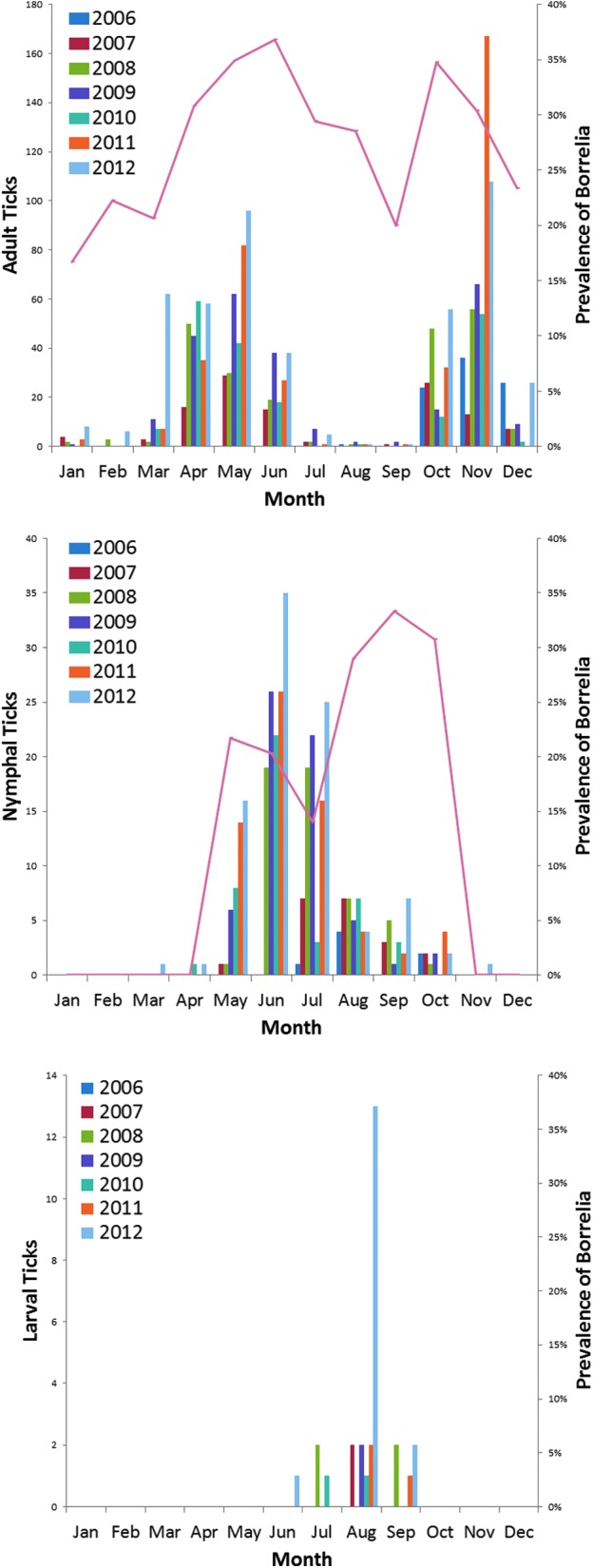
Monthly submission of *I. scapularis* (adults, nymphs, and larvae) and *Borrelia* infection rates from July 2006 through December 2012.

We received *I. scapularis* in all months of the year, although the different life stages exhibited different seasonal activity patterns (Fig. 2). We received 28 larvae between June and September. We received 360 nymphs between April and October, with a clear peak in June. The total of 1700 adults, however, displayed two discrete peaks: the April–June peak representing questing activity of the overwintering population and the October–December peak representing large autumnal populations.

### Age distribution and tick attachment sites of tick bite victims

Of the total 2088 *I. scapularis*, 1962 came from humans, 60 from dogs, 14 from cats, 7 from horses, and 11 from lawns, household floors, or walls. The hosts of the 41 remaining ticks were not reported. The majority (1674) of the 1700 adult ticks submitted were female.

**FIG. 3.**
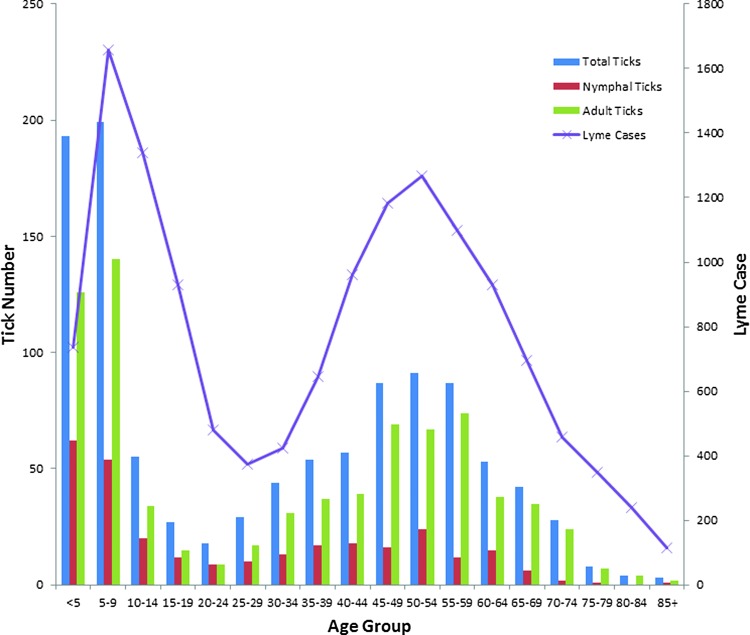
Age distribution of *I. scapularis* bite victims (2006–2012) and Lyme borreliosis cases in Massachusetts (2006–2012). The youngest host age group, individuals aged 0–9 years, had the largest proportion of ticks: 39.7% of the nymphs and 34.6% of the adult ticks. There was a second peak among the 50- to 54-year-olds. People 20 to 24 years old and those over 75 submitted the fewest number of ticks.

Of the *I. scapularis* ticks removed from humans, 47.9% came from men and 52.1% came from women. We found a modal distribution of infestation by host age (Fig. 3). We received age data for 1883 (343 for nymphs and 1540 for adult ticks) of the tick bite victims. The pattern of host age was nearly identical for nymphs and adult ticks. The youngest host age group, individuals aged 0–9 years, had the largest proportion of ticks submitted: 39.7% of the nymphs and 34.6% of the adult ticks. We found a second peak among the 50- to 54-year-olds. People aged 20 to 24 years and those over 75 had the fewest number of reported ticks. The age distribution of hosts in our study mirrors that of the reported cases of Lyme borreliosis in Massachusetts and the United States (Bacon et al. 2008).

We received *I. scapularis* from a wide variety of attachment sites on people (Table 2). Although the number of larvae was too small to compare attachment sites, the attachment sites for nymphs and adult ticks differed strongly (*X^2^* = 49.2, *p* < 0.05). We found that 24.1% of adult ticks and 8.4% of nymphs were attached to the head region, whereas 30.3% of nymphs and 17.0% of adult ticks were attached to the lower extremities. We received very few adult ticks and nymphs that attached to the chest (3.6% and 1.8%, respectively).

**Table 2.** Attachment Sites of *Ixodes scapularis* Adults and Nymphs on Humans

	*Adult ticks*		*Nymphs*	
*Attachment sites*	n	*%*	N	*%*
Abdomen/Groin	223	15.3%	49	14.7%
Buttocks	64	4.4%	14	4.2%
Chest	49	3.6%	6	1.8%
Head	353	24.1%	28	8.4%
Lower extremities	249	17.0%	101	30.3%
Neck	108	7.4%	25	7.5%
Shoulder/Back	230	15.7%	44	13.2%
Upper extremities	186	12.7%	66	19.8%

Lower extremities: thigh, leg, ankle, and foot; upper extremities: arm, forearm, wrist, and hand.

### Prevalence of pathogens in I. scapularis removed from humans

Of 1955 *I. scapularis* ticks found on humans in Massachusetts (2006–2012), 29.6% were infected with *B. burgdorferi*, 4.6% with *A. phagocytophilum*, and 1.8% with *B. microti*. We found that 1.8%, 1.0%, and 0.4% of ticks were coinfected by *B. burgdorferi* and *A. phagocytophilum*, *B. burgdorferi* and *B. microti*, and *A. phagocytophilum* and *B. microti*, respectively. Only 0.3% of ticks had triple coinfection (Table 3).

**Table 3.** Pathogen Prevalence and Coinfection in *Ixodes scapularis* Ticks Found on Humans

*Location in MA*	*County*	*Ticks*	*Bo (%)*	*A (%)*	*Ba (%)*	*Bo+A (%)*	*Bo+Ba (%)*	*A+Ba (%)*	*Bo+A+Ba (%)*
East	Barnstable	282	27.0	5.3	4.6	1.8	2.8	1.1	1.1
East	Bristol	42	33.3	0.0	0.0	0.0	0.0	0.0	0.0
East	Dukes	18	33.3	5.6	11.1	5.6	11.1	5.6	5.6
East	Essex	125	23.2	7.2	5.6	3.2	3.2	1.6	0.8
East	Middlesex	400	33.0	6.8	1.5	3.0	1.0	0.0	0.0
East	Nantucket	8	62.5	12.5	0.0	12.5	0.0	0.0	0.0
East	Norfolk	94	27.7	4.3	0.0	1.1	0.0	0.0	0.0
East	Plymouth	121	20.7	2.5	2.5	1.7	0.0	0.0	0.0
East	Suffolk	17	11.8	17.6	5.9	0.0	0.0	5.9	0.0
Central	Worcester	222	33.8	5.9	0.0	2.7	0.0	0.0	0.0
West	Berkshire	112	32.1	2.7	0.9	0.0	0.0	0.0	0.0
West	Franklin	234	29.5	3.0	0.0	0.9	0.0	0.0	0.0
West	Hampden	35	11.4	0.0	0.0	0.0	0.0	0.0	0.0
West	Hampshire	245	32.2	1.6	1.2	0.4	0.8	0.0	0.0
Total		1955	29.6	4.6	1.8	1.8	1.0	0.4	0.3

A, *Anaplasma phagocytophilum*; Ba, *Babesia microti*; Bo, *Borrelia burgdorferi.*

## Discussion

Our passive surveillance provides valuable data for assessing the risk of human exposure to tick-borne diseases. It directly measures linkages between ticks, tick-borne pathogens, and tick bite victims. The risk of *B. burgdorferi* transmission is determined by a confluence of at least three key factors: (1) chance of encountering ticks, (2) infection status of the ticks, and (3) duration of tick bites. Each of these factors is necessary for transmission of tick-borne infection, but no single factor is sufficient by itself. Traditional field studies involving tick flagging surveillance or canine serosurveys will, at best, indirectly provide an approximation of only two of the three key factors. Passive surveillance provides measures all three factors of risk.

Passive surveillance of tick-borne disease has been done elsewhere. Expanding range and proliferation of *I. scapularis* prompted passive tick surveillance in Canada in the 1990s (Ogden et al. 2006). Koffi et al. (2012) pointed out that passive surveillance based on sampling of human-biting ticks lacks power to detect the risk of tick-borne disease. Confounding factors of host and tick dispersal and behavior make it difficult to determine where tick populations have established and are locally reproducing (Koffi et al. 2012). The passive surveillance outlined in the present article is not subject to this bias since *I. scapularis* populations are endemic and locally reproducing throughout the state of Massachusetts, allowing us to assess geographic and temporal distributions of ticks and prevalence of tick-borne pathogens in human-biting ticks. Combining passive tick-borne disease surveillance data with human population and environmental variables can provide even more valuable information for detection of tick-borne disease risk.

Prior passive surveillance studies have shown that *D. variabilis* slowly expanded in Maine (Rand et al. 2007). The increasing numbers of *I. scapularis* submissions may indicate growing local tick populations; however, they also could be an artifact of our passive sampling scheme or because of increasing human population density and activity. Our results suggest that tick populations in Massachusetts have a patchy discontinuous distribution with four major areas of high density. The broadly defined high-density areas are Cape Cod, the eastern half of the state outside of Suffolk county, parts of Franklin and Hampshire counties along the Quabbin Reservoir watershed, and in southwestern Berkshire county (Fig. 1). The high tick encounter areas are also areas of high Lyme borreliosis incidence. Our results as well as results from a study conducted in New Hampshire (Walk et al. 2009) show that Lyme borreliosis is more prevalent in areas of high tick density, suggesting that tick pathogens are more prevalent among long-established tick populations than among recently established populations.

Information on duration of tick feeding, attachment site, and victim age provides valuable insights for tick-borne disease prevention. A favorable outcome following a tick encounter (*i.e*., no disease transmission) depends on removal of the tick within 24–48 h (Piesman et al. 1987, des Vignes et al. 2001). Tick engorgement status is an important component of disease transmission risk assessment. In our study of human-biting ticks, we found an overall trend for decreasing engorgement percentage of larvae, nymphs, and adults: 65.0%, 50.9%, and 35.1%, respectively. This result is likely due to the increasing body size of each successive stage of development; the larger the tick, the larger it will be when engorged, and hence the more likely it will be detected and removed before repletion (Yeh et al. 1995). Nonetheless, the duration of tick feeding varies markedly among victim age groups. The rate of engorged nymphs attaching to children younger than 9 years was only 35.6%, which was significantly lower than for other age groups. For most victims over the age of 20 who were bitten by ticks, rates of finding engorged nymphs and adult ticks increased with age. Ticks attacking victims over the age of 75 were less likely to be removed before becoming engorged. Overall, these data suggest behavioral trends in tick-checking activity: younger children (more likely their parents) are doing the best job, adults should pay more attention to checking for nymphal ticks, and seniors are the least adept at finding and removing both nymphal and adult ticks.

The available information on tick attachment sites is inconsistent. While Felz and Durden (1999) found no apparent preference for attachment sites on humans in Georgia and South Carolina, Falco and Fish (1988) studied the attachment sites of *I. scapularis* in New York and found that nymphs preferred the lower extremities, while adult ticks preferred the head. We found *I. scapularis* attachment sites distributed throughout almost all of the body sites, with significant differences between nymphs and adult ticks. Nymphs were more likely to be attached to lower extremities, while adult ticks were more frequently found attached near the head, followed by the lower extremities. This may be biased because ticks on the legs and head are most easily detected. In victims aged 9 years and under, 49% and 13% of adult ticks attached to the head and the neck, respectively. Therefore, examination of the head and neck is most important during the adult tick season in autumn and early spring, while during late spring and summer, careful examinations of the lower extremities are more crucial because of the small size of the nymphal-stage ticks active during these seasons.

Previous results concerning gender-specific risk for Lyme borreliosis are inconsistent. In most cases, males have a higher risk (Bacon et al. 2008), although a study on Nantucket Island found females at higher risk (Phillips et al. 2001). We did not find any gender-specific risk differences among the tick bite victims.

It is well known that *I. scapularis* can simultaneously or sequentially infect their hosts with *B. burgdorferi*, *A. phagocytophilum*, and *B. microti* (Holman et al. 2004, Swanson et al. 2006). However, it is not well understood where and how often coinfection occurs. Lack of reliable methods for coinfection detection and quantification is one of the reasons. Our report includes a description of two duplex real-time PCR assays to quickly and simultaneously identify three common tick-borne pathogens. Our results show that all three pathogens are present in Massachusetts; however, their geographic distribution and tick infection rate are quite different. First, *B. burgdorferi* has been found all over Massachusetts and is more widespread geographically than either *A. phagocytophilum* or *B. microti*. The prevalence of *B. burgdorferi* in nymphs (20.8%) and adults (32.0%) is relatively uniform among four high tick density areas in Massachusetts. (Table 3). The number of reported Lyme borreliosis cases and ticks has increased in recent years; however, the results of this study and our previous field study (Walk et al. 2009) suggest that the prevalence of *B. burgdorferi* among ticks is relatively stable year to year. Second, unlike Lyme borreliosis, the majority of *A. phagocytophilum* (77.7%) and *B. microti* (89.5%)-infected ticks are currently geographically limited to Cape Cod and the eastern half of Massachusetts. The average statewide infection rates of *A. phagocytophilum* (4.6%) and *B. microti* (1.8%) are lower than they are on Nantucket Island (Telford et al. 1996). *A. phagocytophilum* and *B. microti* are present in western Massachusetts with low prevalence. Third, the low rate (0.3–1.8%) of coinfection of *B. burgdorferi*, *A. phagocytophilum*, and *B. microti* was found mainly in ticks from eastern Massachusetts. The triple coinfection was only found in adult ticks from eastern coastal locations (Table 3). Coinfection of *A. phagocytophilum* and *B. burgdorferi* increases the clinical impact of both pathogens and results in more severe Lyme arthritis symptoms (Grab et al. 2007). The medical importance of coinfection by *B. burgdorferi*, *A. phagocytophilum*, and *B. microti* should be evaluated in regions where three pathogens are endemic.

Our passive surveillance may also serve as an early warning system for ticks and tick-borne pathogens. For example, the geographic range of the Lone Star tick, *A. americanum*, is reportedly expanding northward (Keirans and Lacombe 1998). Based on the seven *A. americanum* ticks that we identified from western Massachusetts, we consider this tick a residential but occasional species in Massachusetts. Although the majority of *A. phagocytophilum* and *B. microti* are in eastern Massachusetts, these pathogens are present in low infection rates in western Massachusetts. Will some tick species and pathogens spread quickly in New England as seen in *I. scapularis* and Lyme disease? A continuous, passive tick surveillance can monitor changing trends in local population density and risk for disease outbreaks.

## Conclusions

Prevention is the key for minimizing tick-borne diseases. Two separate duplex real-time PCRs can be used for passive tick surveillance on a statewide scale to identify high-risk areas by resolving geographic, temporal, and behavioral distributions of ticks and the pathogens they carry. Because our data on tick bite incidences corroborate known epidemiological patterns of Lyme borreliosis, we maintain that passive surveillance surveys are important tools for detecting and monitoring established and emerging tick-borne pathogens.
